# Rab GTPases as regulators of endocytosis, targets of disease and therapeutic opportunities

**DOI:** 10.1111/j.1399-0004.2011.01724.x

**Published:** 2011-10

**Authors:** JO Agola, PA Jim, HH Ward, S BasuRay, A Wandinger-Ness

**Affiliations:** a Department of Pathology, University of New Mexico School of MedicineAlbuquerque, NM 87131, USA; b Cancer Center, University of New Mexico School of MedicineAlbuquerque, NM 87131, USA

**Keywords:** cancer, Charcot-Marie-Tooth disease, GTPase networks, mental retardation, neurodegenerative disease, neuropathies, pigmentation, immune and bleeding disorders

## Abstract

Rab GTPases are well-recognized targets in human disease, although are underexplored therapeutically. Elucidation of how mutant or dysregulated Rab GTPases and accessory proteins contribute to organ specific and systemic disease remains an area of intensive study and an essential foundation for effective drug targeting. Mutation of Rab GTPases or associated regulatory proteins causes numerous human genetic diseases. Cancer, neurodegeneration and diabetes represent examples of acquired human diseases resulting from the up- or downregulation or aberrant function of Rab GTPases. The broad range of physiologic processes and organ systems affected by altered Rab GTPase activity is based on pivotal roles in responding to cell signaling and metabolic demand through the coordinated regulation of membrane trafficking. The Rab-regulated processes of cargo sorting, cytoskeletal translocation of vesicles and appropriate fusion with the target membranes control cell metabolism, viability, growth and differentiation. In this review, we focus on Rab GTPase roles in endocytosis to illustrate normal function and the consequences of dysregulation resulting in human disease. Selected examples are designed to illustrate how defects in Rab GTPase cascades alter endocytic trafficking that underlie neurologic, lipid storage, and metabolic bone disorders as well as cancer. Perspectives on potential therapeutic modulation of GTPase activity through small molecule interventions are provided.

The Ras superfamily of GTPases governs trafficking, cytoskeletal regulation and cell signaling, through the interdigitated functions of Rab, Arf/Arl, Rho, Ras and Ran subfamily members. In this review we focus on the Rab GTPases that function in endocytosis and recycling, phagocytosis, autophagy and pinocytosis (Fig. 1, left side). As noted in Fig. 1, endocytic Rab GTPases also play vital roles in lysosome biogenesis and ciliogenesis reviewed in Refs. (1–3). We contrast normal function to the consequences of Rab GTPase or accessory protein dysfunction in genetic or acquired diseases (Fig. 1, right side). Using pathway specific examples we provide information on the mechanistic underpinnings of normal function and alterations that lead to disease. Mutations in the Rab GTPases and accessory proteins themselves, altered GTPase expression or activity are relevant to neurologic and neurodegenerative diseases, lipid storage disorders and cancer. The pivotal roles of small GTPases in disease make them attractive targets for therapeutic intervention that have not yet been broadly explored. We also illustrate the fundamental aspects for GTPase regulation that provide possible paradigms for small molecule therapeutics.

**Fig. 1:**
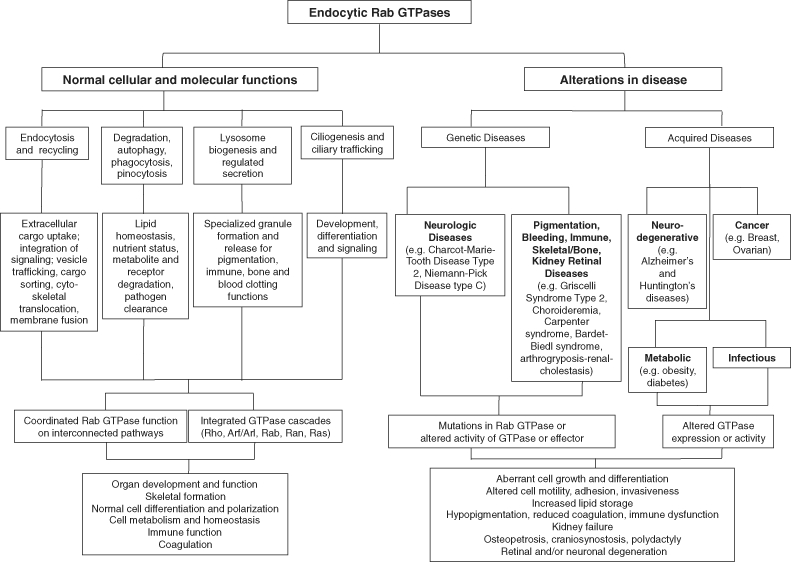
*Rab GTPases in normal cellular and molecular functions vs alterations in human disease.* (Left panel) Normal functions of endocytic Rab GTPases in (i) endocytosis and recycling, (ii) degradative pathways (including autophagic and phagocytic pathways), (iii) lysosome biogenesis and regulated secretion, (iv) ciliogenesis and ciliary trafficking. (Right panel) Disease-causing alterations in Rab GTPase function may be acquired or genetic leading to loss of function, increased or decreased expression or activity.

## Rab GTPase regulated endocytosis, recycling and signaling

**Fig. 2:**
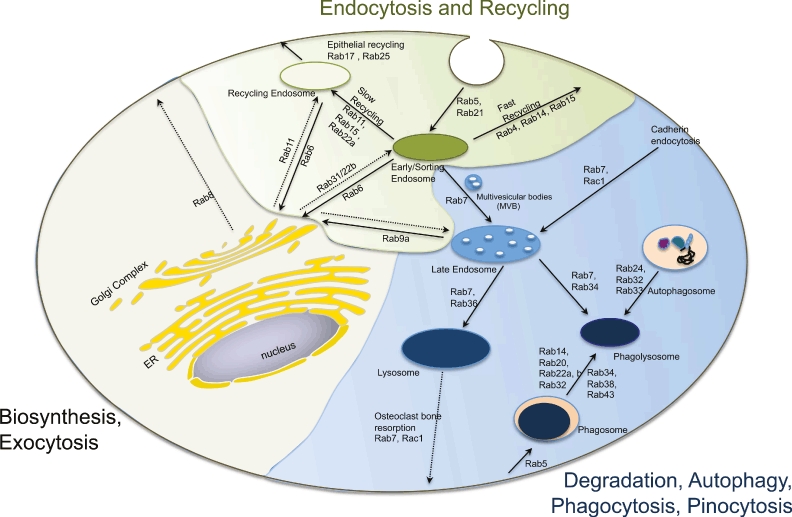
*Rab GTPases in endocytosis, recycling, and degradative pathwa*y*s*. Receptor mediated endocytosis occurs via clathrin-coated vesicles and is regulated by Rab5 and Rab21. Internalized cargo is delivered to early/sorting endosomes. From here molecules can return to the plasma membrane via fast or slow recycling routes through specialized recycling endosomes and the activities of distinct Rab GTPases (further detailed in the text). Newly synthesized plasma membrane proteins are delivered from the trans-Golgi network to recycling endosomes, while lysosomal hydrolases are delivered to early and late endosomes via two mannose 6-phosphate receptors. Recycling from early endosomes to the Golgi depends on Rab6, while Rab9 controls transport from the late endosome to the trans-Golgi. Rab7 is a critical Rab GTPase on multiple degradative pathways; promoting late endosome, phagosome and autophagosome fusion with lysosomes in cooperation with specialized Rab GTPases on each of these pathways. In conjunction with Rac1, Rab7 is also pivotal in cadherin degradation by epithelia and neurons, as well as in bone resorption by osteoclasts. Light green overlay encompasses endocytic and recycling circuits; light blue overlay encompasses degradative circuits; and neutral overlay encompasses the biosynthetic/exocytic routes.

Among the over 70 mammalian Rab GTPases (4), there are ∼20 Rab proteins with defined functions in endocytosis and related processes (Table 1). Early endocytic and recycling routes are summarized in Fig. 2 (green section). Receptor-mediated endocytosis occurs via clathrin-coated vesicles and is principally regulated by Rab5 and Rab21, sharing common regulatory and effector proteins (5, 6). Following internalization, sorting in the early endosome segregates molecules for return to the plasma membrane along fast (Rab4, Rab14, Rab15) and slow (Rab11a, Rab15, Rab22a) recycling routes (7–11). Epithelia have specialized recycling circuits that utilize Rab17 and Rab25 in addition to the ubiquitous Rab4, Rab5 and Rab11 GTPases (12). Early endosomes also regulate return to the Golgi via Rab6 and the retromer complex, which may also interact with Rab7 (13, 14). From late endosomes, return to the Golgi is regulated by Rab9, STX10 and Tip47 (15). Thus, endosomes and associated Rab GTPases serve in cargo sorting for plasma membrane and Golgi recycling, as well as for degradation.

**Table 1:** Rab GTPase functions, networks and disease associations^a^,^b^

Rab	Localization	Rab function	Pathological condition
Endocytosis and recycling			
Rab4a	Early endosomes and recycling endosomes	Regulates sorting and endocytic recycling to the plasma membrane	Upregulated in rodent model of diabetic cardiomyopathy, human systemic lupus erythematosus, Alzheimer's disease and Down's syndrome; inhibited in Niemann–Pick disease; downregulated in tumor cells
Rab5a	Plasma membrane, clathrin-coated vesicles and early endosomes	Endocytosis, early endosome fusion, nuclear signaling through APPL	Hyperactivated in lung adenocarcinoma; upregulated in Alzheimer's Disease
Rab9a	Late endosomes	Transport from endosome to TGN; lipid transport; lysosome and lysosome-related organelle biogenesis	Inhibited in Niemann–Pick C disease
Rab11a, Rab11b (neuron specific)	Golgi and recycling endosomes, early endosomes, phagosomes	Trafficking from the TGN to apical recycling endosomes and plasma membrane; polarized trafficking in epithelia; phagocytosis in macrophages	Upregulated in Barrett's epithelia; neurodegeneration in Huntington's disease; Schwann cell demyelination in Charcot-Marie-Tooth type 4C disease; implicated in Batten disease
Rab14	Early endosome, Golgi	Endocytic recycling of transferrin; MHC class I cross-presentation; TGN to apical trafficking in epithelia; surfactant secretion in alveolar cells; insulin-dependent GLUT4 translocation	
Rab15	Early/sorting endosome, recycling endosome	Trafficking through sorting/recycling endosomes to the plasma membrane	
Rab17	Recycling endosome	Epithelial transcytosis; polarized trafficking in kidney	
Rab20	Phagosomes, mitochondria, endosomes	Vacuolar ATPase trafficking in kidney; HIF target in hypoxia induced apoptosis; phagosome acidification and maturation; Gap junction biogenesis	Modulated by pathogens; overexpressed in pancreatic and breast cancers
Rab21	Early endosomes; macropinosomes	Endocytosis of integrins, cell extracellular matrix adhesion and motility; cytokinesis; macropinocytosis	Cancer cell motility
Rab22a	Early endosome, plasma membrane	Transport of transferrin from sorting endosomes to recycling endosomes; pathogen phagocytosis	Upregulated in hepatocellular carcinoma; modulated by mycobacterium tuberculosis
Rab25	Recycling endosome	Apical recycling in epithelia, microtubule dependent transformation	Tumor progression and cancer invasiveness (breast and intestinal cancers; ovarian cancer and hepatocellular carcinoma)
Rab31/Rab22b	TGN and endosomes	Mannose-6-phosphate transport from TGN to endosomes; transport of myelination associated proteins from TGN to plasma membrane	
Rab34	Golgi and endosomes	Macropinosome formation, phagosome maturation and lysosome morphogenesis	Diabetic nephropathy
Rab35	Endosomes and plasma membrane	Fast endocytic recycling; MHC class I and II endocytosis and recycling; Tcell receptor recycling; phosphoinositide regulation; neurite outgrowth through interfaces with Cdc42; actin remodeling through fascin effector	Pathogen phagocytosis and trafficking
Rab36	Golgi	Late endosome and lysosome clustering	Potential tumor suppressor
Rab39	Golgi and early endosomes, AP1 membrane domains	Caspase-dependent-IL-1*β* secretion and phagosomal acidification	
Autophagy, phagocytosis and degradation			
Rab7a	Late endosomes and lysosomes; stage I and II melanosomes; surfactant endocytosis and signaling	Transport from early to late endosomes and late endosome to lysosome fusion; bidirectional transport of signaling endosomes, autophagosomes, and multivesicular bodies on microtubules in association with dynein and kinesin motor proteins. Axon viability; phosphoinositide homeostasis	Mutant in CMT2B; Helps in pathogen entry and survival; associated with Niemann–Pick disease; upregulated in Alzheimer's disease, thyroid cancer, diffuse peritoneal malignant mesothelioma and adult-onset obesity
Rab24	Autophagosome nuclear inclusions	Myelination; autophagosome formation	Activated in cell culture models of neuronal and cardiomyocyte injury; upregulated in hepatocellular carcinoma
Rab32	Perinuclear vesicles, mitochondria, autophagic vesicles	Post-Golgi trafficking of melanogenic enzymes; ER stress mediated apoptosis; mitochondrial dynamics	*Rab32* gene methylated in inflammatory bowel disease at transition to invasive growth

CMT2B, Charcot-Marie-Tooth Disease Type 2B; TGN, trans-Golgi network.

a Source: Agola JO, Thesis.

b Rab GTPases are clustered according to their functions in: (i) endocytosis and recycling; (ii) degradation, autophagy, phagocytosis and pinocytosis.

The early internalization and recycling circuits are subject to up- or downregulation in response to signaling and cellular demand. For example, epidermal growth factor receptor (EGFR) signaling through Ras increases the activity of Rin1 whose guanine nucleotide exchange factor (GEF) activity causes Rab5 association with signal-transducing adaptor molecule 2 (STAM2) to increase EGFR internalization and degradation (16). Rab11a activation on the other hand increases EGFR recycling in response to EGF signaling and the Rab5 GTPase activating protein (GAP), RN-TRE, decreases EGFR endocytosis through Grb2 (17). Each of the latter two pathways results in greater EGFR at the plasma membrane in lieu of degradation. Rab5, Rab11, Rab21 and Rab25 are not only important regulators of growth factor signaling and downregulation, but also regulate cell adhesion and motility through targeted insertion and recycling of integrins (6). The selected examples emphasize the importance of pathway integration in the coordination of trafficking, signaling, adhesion and migration.

Besides regulating the fate of cell surface receptors, early endosomes also serve as platforms for the assembly of endosome-specific complexes critical to nuclear signaling and transcriptional regulation (18). For example, EGFR is sequestered in Rab5 positive early endosomes together with the Rab5 effector APPL1, promoting nuclear translocation of APPL1 and changes in gene expression (19). In neurons, retrograde transport of the nerve growth factor signaling complexes via endosomes is essential for axon viability through Erk signaling and transcriptional regulation. This pathway is negatively regulated by the Rab5 effector huntingtin, which increases endosome links to the peripheral actin network and reduces nuclear signaling (20). As illustrated, Rab-regulated trafficking and endosomal signaling is directly linked to gene regulation.

## Rab GTPase roles in degradation, autophagy, phagocytosis and pinocytosis

Endocytosed and phagocytosed cargo destined for degradation are transferred from early to late endosomes and require fusion with lysosomes in a Rab7-dependent process (Fig. 2, blue section) (21). Sequestration of cellular organelles and cytoplasm in response to nutrient limitation is regulated through autophagosome formation and a Rab7-regulated convergence with the endolysosomal system (22). Rab7 together with Rac1 is involved in cell type specific functions including the regulation of cell–cell adhesion through the degradation of cadherins in epithelia and neurons as well as osteoclast-mediated bone resorption regulating cell–cell adhesion (23). These examples illustrate the complexity of the late endosomal circuits.

Trafficking and cargo sorting (including membrane invagination and receptor sequestration) on the early and late endocytic pathway require spatially and temporally regulated phosphoinositide synthesis and degradation, which are closely linked to Rab5 and Rab7 GTPase activities (24, 25). The activated GTPases in combination with PtdIns(3) P-enriched domains enable binding of Rab GTPase effectors bearing FYVE domains (Rab5 EEA1; Rab7 FYCO) or PX domains (sorting nexins SNX) and cargo recognition factors like retromer (26). Lipid phosphatases serve to terminate the lipid-signaling cascade. The endosomal lipid phosphatases MTM1 and MTMR2 responsible for degrading PtdIns(3)P and PtdIns(3,5)P_2_ bind to the hVps15/hVps34 lipid kinase causing the inactivation of both kinase and phosphatase activities (27). The late endosomal PIKFyve (PIP5K3) lipid kinase produces PtdIns(5)P/PtdIns(3,5)P_2_ and associates with Rab9 via the p40 protein to regulate late endosome to trans-Golgi transport of the mannose 6-phosphate receptor (28). The activity of PIKFyve is counterbalanced by the FIG4 PtdIns(3,5)P_2_ 5-phosphatase and thought to be involved in melanosome and neurosecretory granule formation (1). The cumulative data show dynamic regulation of endosomal phosphoinositide metabolism is central to Rab-mediated trafficking.

Autophagy, phagocytosis, and pinocytosis are specialized endocytic cellular processes regulated by multiple Rab GTPases (Fig. 2). Phagocytosis and pinocytosis are crucial for the uptake of small particles and bacterial pathogens by cells of the immune system, while autophagy is a ubiquitous housekeeping function vital for the autodigestion of cell constituents and pathogen clearance (29). Overlapping GTPase subsets work in concert to regulate phagosome (Rab14, Rab20, Rab22a, b, Rab32, Rab34, Rab38, Rab39, Rab43), macropinosome (Rab21, Rab34) and autophagosome (Rab24, Rab32, Rab33) formation (30, 31). For example, degradative enzyme delivery to phagosomes and autophagosomes occurs through a common mechanism involving fusion with endo/lysosomes and is regulated by Rab7 (32). Whether it is endocytosis, autophagy or phagocytosis, the roles of Rab GTPase are tightly regulated by interactions with partner proteins that ensure the integrity of these vital processes.

## Regulation of endocytic Rab GTPases

**Fig. 3:**
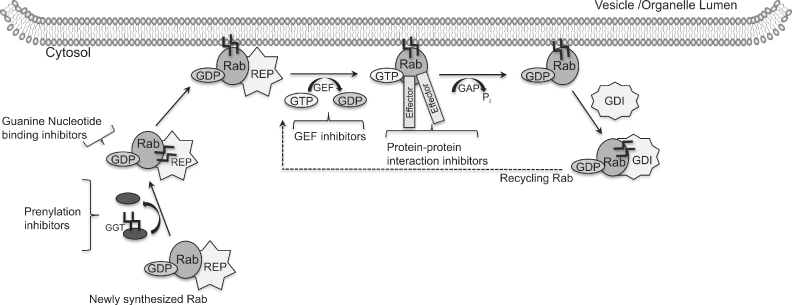
*Rab GTPase regulation and points of therapeutic intervention*. The Rab GTPase activation cycle entails lipid modification through prenylation, nucleotide binding and hydrolysis regulated by guanine nucleotide exchange factors (GEFs) and GTPase activating proteins (GAPs), effector protein interactions and cytosolic recycling by guanine dissociation inhibitor (GDI). Possible modes of Rab GTPase inhibition include disruption of membrane association through prenylation inhibitors; GEF inhibitors to block activation; nucleotide binding inhibitors to block activation analogous to kinase inhibitors; and protein–protein interaction inhibitors.

Principal processes pertinent to Rab regulation include prenylation and membrane insertion, nucleotide binding and hydrolysis, and effector protein interaction (Fig. 3) (4). Figure 3 also shows points of therapeutic intervention that will be discussed in a later section. Rab proteins are initially synthesized as soluble proteins in the cytosol (33), where they are recognized by the Rab escort protein (REP) and presented to the Rab geranylgeranyl transferase enzyme for the addition of one or two geranylgeranyl lipid groups to *C*-terminal cysteine residues (34). Prenylation together with the hypervariable *C*-terminus of the Rab protein ensures stable and targeted membrane insertion (35). On the target membrane, specific GEFs convert Rab proteins from the GDP-bound state into the GTP-bound state (36).

Active Rab GTPases interact temporally and spatially with many effector molecules; including sorting adaptors, tethering factors, kinases, phosphatases and motor proteins that ensure cargo sorting, vesicle transport and fusion (37). Rab GTPase inactivation occurs through intrinsic GTPase hydrolysis and GAP-stimulated catalysis (38). Recycling of the GDP-bound Rab GTPase occurs through cytosolic recycling mediated by guanine dissociation inhibitor (GDI). The whole Rab GTPase regulatory process is conceptually analogous to an electronic circuit system in which GEFs taking Rabs to the active state can be viewed as electronic signal amplifiers, while GAPs that inactivate Rabs can be considered as signal attenuators. Effector molecules that couple Rab proteins with downstream signaling events are analogous to electronic signal integrators.

## Alterations in endocytosis leading to disease

Numerous Rab GTPases on the endosomal and autophagy circuits described in the previous sections are implicated in acquired and genetic diseases through up- or downregulation or modulation of their activities (Table 1). Such imbalanced increases or decreases in GTPase activity and expression alter cell physiology, affect gene expression and hence contribute to disease pathology as we discuss further below.

**Fig. 4:**
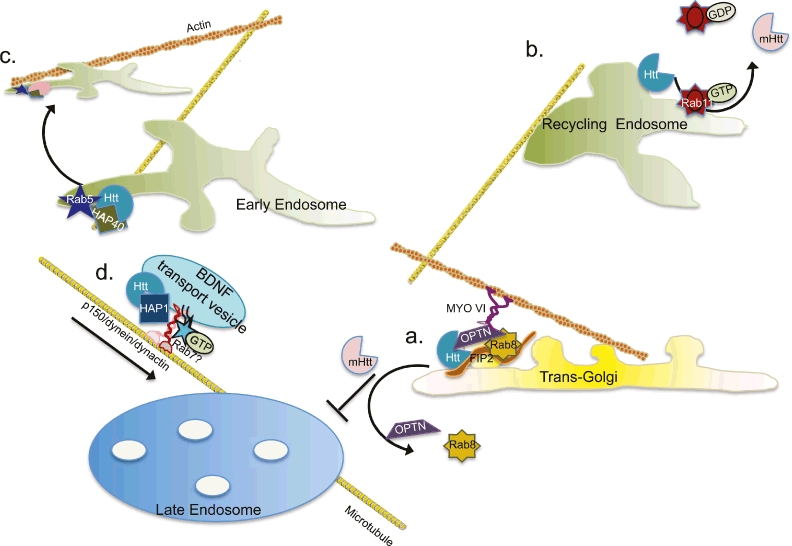
*Genetic diseases disrupt endocytic recycling circuits and cause neuronal degeneration, glaucoma and retinal degeneration*. The schematic depicts the normal functions and protein complexes of the Huntingtin (Htt) and optineurin (OPTN) proteins on the Golgi and various endosomal compartments. Mutant Htt (mHtt) causes the severe neurodegenerative Huntington's disease and mutant OPTN causes vision disorders. (**a**) Htt protein is associated with the trans-Golgi and post-Golgi vesicles where it binds OPTN, Fip2, Rab8 and myosin VI. mHtt expression causes OPTN and Rab8 dissociation and blocks lysosomal enzyme export to endosomes. (**b**) Htt increases guanine nucleotide exchange factor (GEF)-mediated activation of Rab11a on recycling endosomes. mHtt causes Rab11 inactivation and release from endosomes. (**c**) Htt binds HAP40 and Rab5 on early endosomes. mHtt causes early endosome transfer from microtubules to actin filaments and peripheral immobilization. (**d**) Htt forms a complex with HAP1, the p150dynein/dynactin motor complex that is important in trafficking brain-derived neurotrophic factor (BDNF) containing vesicles to late endosomes.

Defects in rab regulatory factors lead to disease by perturbing Rab GTPase membrane localization or phosphoinositide metabolism and consequently impinging on Rab activity. Choroideremia *(CHM)* is an X-linked form of retinal degeneration caused by loss of function mutants in REP-1 thereby reducing membrane association (39). Conversely, some patients diagnosed with X-linked non-specific mental retardation have mutations in the *GDI1* gene that preclude proper Rab recycling after vesicle fusion (40). Lipid kinases and phosphatases central to rab-regulated trafficking are targets of disease-causing mutations in humans (Lowes and Dents –*OCRL1*, corneal dystrophy –*PIKFyve*, ALS and Charcot-Marie-Tooth disease – (CMT4J), neurologic and pigmentation disorders –*FIG4* and kidney disease –*INPP5E*, *OCRL1*) (41–44).

Genetic mutations associated with Rab5 activation have been linked to pathogenesis of genetic disorders like Ehlers-Danlos and the cutis laxa syndromes (45). These connective tissue disorders are caused by mutant Rab5-GEF RIN2 and are clinically manifested by progressive facial coarsening, gingival hypertrophy, severe scoliosis, sparse hair and skin and joint hyperlaxity (45, 46). At the molecular level, impaired Rab5-dependent trafficking results in decreased secretion of skin microfibrils and fibulin-5 that are crucial for tissue elasticity (46).

Amyotrophic lateral sclerosis 2, an autosomal recessive form of motor neuron disease is associated with mutations in the *ALS2* gene (47, 48). ALS2/alsin is another Rab5 GEF important in neuronal endocytosis and neurite outgrowth (49). Mutations in *ALS2* gene give rise to truncated forms of alsin that increase glutamate receptor degradation and alter autophagy (50). Altered Rab5 GEF activities may affect Rab5–Rab7 conversion. Support for Rab7 involvement is garnered from the accumulation of enlarged APP/Rab7 positive vesicles in the mouse mutant wobbler (WR), a model of sporadic amyotrophic lateral sclerosis (51). Thus, connective tissue and neuronal degeneration caused by mutations in distinct Rab5 GEFs may affect both Rab5 and Rab7, as well as downstream degradative pathways.

Neurodegenerative diseases and vision defects may arise by impacting common effector proteins (52). For example, inherited Huntington's disease is a devastating neurologic disease caused by the expression of mutant Huntingtin (mHtt) proteins with long polyglutamine tracts at their *N*-termini. The altered proteins cause the accumulation of abnormal intracellular aggregates and inclusions. Clues regarding Htt function can be surmised from studies of its interactions with several exocytic and recycling Rab GTPases (Fig. 4) (53–55). As illustrated, Htt is a critical scaffolding protein. Normal Htt function promotes Rab activation and motor protein assemblies that drive vesicular transport on actin and microtubule cytoskeletal networks, in part explaining how loss of Htt function significantly affects neuronal function. The Htt interacting protein, optineurin, causes open angle glaucoma when mutant.

Genetic lipid storage disorders such as the neurodegenerative Batten disease and Niemann–Pick type C disease are caused by Rab GTPase inactivation by cholesterol accumulation due to impaired lysosomal hydrolase trafficking (56, 57). Mutation of the *CLN3* gene, which encodes an endosomal/lysosomal transmembrane protein, causes Batten disease (56). CLN3 protein forms a complex with the microtubule binding protein Hook1/Btn2p and several Rab GTPases (Rab7, Rab9 and Rab11) (56). Ablation of CLN3 results in mannose 6-phosphate receptor accumulation in the Golgi and reduction in maturation of lysosomal enzymes (58). These data provide evidence that positive benefit may be provided in multiple lipid storage diseases through increasing Rab7 and Rab9 protein expression and/or activity.

**Fig. 5:**
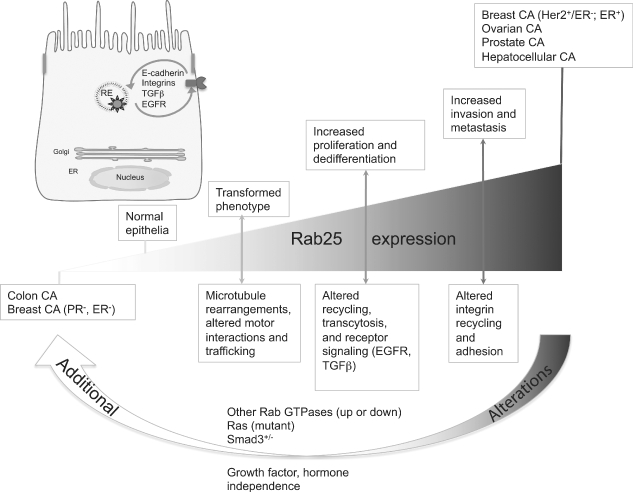
*Rab GTPase up- or downregulation can result in acquired disease.* Rab25 serves as a well-studied example of how increased or decreased GTPase expression can impact trafficking of critical molecules involved in cell signaling and adhesion and adversely affect cell physiology in cancer by increasing proliferation, dedifferentiation, motility and metastasis. It is speculated that in cases where Rab25 expression is decreased (e.g. colon and some types of breast cancer) that additional mutations or factors may be in play that are either synergistic with Rab25 underexpression or result in independence from proteins/functions regulated by the Rab25 recycling route.

Rab25 is an illustrative example of altered expression that is closely associated with multiple solid tumor types; this includes ovarian, breast, prostate and intestinal carcinomas (59). Rab25 is an epithelial-specific GTPase operative on the apical recycling circuit with Rab11a and important in apical to basolateral transcytosis (60). Both Rab25 and the two Rab11 isoforms share effectors involved in cargo recognition and motor proteins (FIP1-3, RIP11 and myosinVb) (61). In ovarian cancer, Rab25 mRNA and protein expression are significantly upregulated due to gene amplification (1q22) and strongly correlated with cell invasion and metastasis (Fig. 5) (62). Overexpressed Rab25 results in the transformation of normal intestinal epithelia and depends on microtubule rearrangements, as well as microtubule modifications that affect Rab25/MyoVb-dependent cargo recycling (63). Rab25 and Rab11a are implicated in EGFR and TGF*β* signaling and trafficking with important impacts for the regulation of cell proliferation and differentiation (64, 65).

The role of Rab25 in metastasis is based on its interaction with integrin *α*5*β*1 to promote regulated insertion at the tips of pseudopods, facilitating extracellular matrix adhesion and penetration by invading tumor cells (66). Thus, imbalances in Rab25-regulated processes can affect normal epithelial function and lead to transformation. There, however, remains debate as to why Rab25 is over expressed in some cancers and absent in others. It is speculated that in early stages of transformation Rab25 overexpression is a universal driving factor as illustrated by overexpression studies of Rab25 in intestinal epithelia (Fig. 5). Subsequently, tumorigenesis in some cases becomes independent of Rab25 function either due to coordinated upregulation of other recycling GTPases as is seen in hepatocellular carcinoma (Table 1) or due to additional alterations in signaling pathways and receptors as suggested by colon cancer animal model studies and differences in breast cancer that are correlated with grade (Fig. 5).

Alteration of Rab GTPase function through direct mutation is best illustrated by Charcot-Marie-Tooth type 2B (CMT2B) disease; a peripheral neuropathy with axonal degeneration linked to four separate missense mutations in *Rab7* gene (67, 68). The mutations target highly conserved residues that reside on the surface of Rab7 and alter nucleotide exchange (68, 69). In neuronal N2A and PC12 cell lines, mutant Rab7 expression inhibits neurite outgrowth. Such changes in neuronal differentiation are likely due to prolonged phosphorylation of the TrkA receptor and altered Erk nuclear signaling (68).

**Fig. 6:**
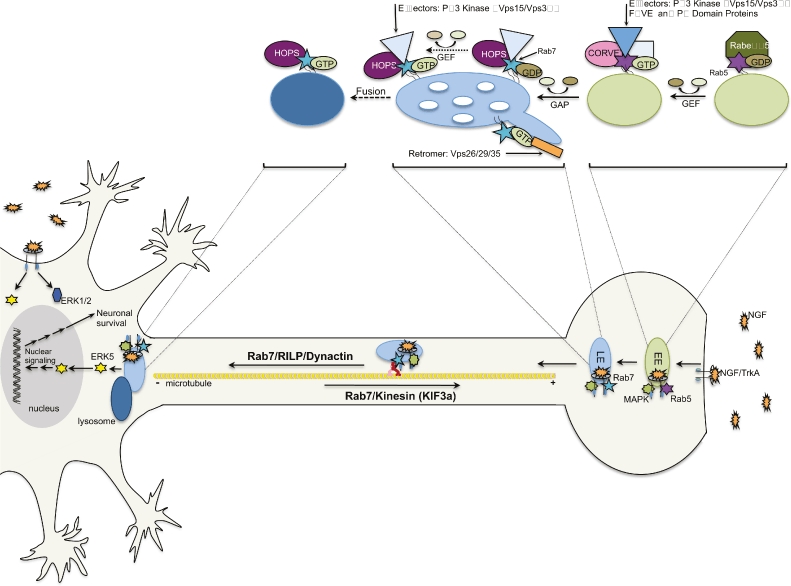
*Axon viability depends on coordinated endocytic trafficking and signaling*. Nerve growth factor (NGF) binds to the TrkA receptor tyrosine kinase and stimulates phosphorylation and internalization. Endocytosed TrkA is transported from Rab5-positive early endosomes to Rab7-positive late endosomes. In peripheral neurons long distance transport of late endosomes to the cell body is critical for growth factor degradation and proper nuclear signaling to maintain cell viability and differentiation. Transport occurs on microtubules through Rab7, the Rab interacting lysosomal protein (RILP) effector and dynactin minus-end directed motor complex. Return transport to the synapse is mediated by Rab7 in conjunction with a plus-end directed kinesin motor, likely KIF3a. Inset illustrates endosomal membrane protein complexes involved in Rab conversion that allows transfer of cargo along the degradative pathway. Several conserved multimeric protein complexes first identified in yeast are thought to aid in Rab conversion, directed transport and fusion (RETROMER endosome to Golgi; CORVET early to late endosome; HOPS late endosome to lysosome).

Similar to ALS2, CMT2B mutant proteins may alter Rab5–Rab7 conversion, Rab7–effector protein interactions and impact the Rab7-regulated trafficking required for maintenance of axon viability (Fig. 6). For example, the CORVET and HOPS protein complexes identified in yeast and conserved in mammalian systems are thought to enable transition of cargo from early to late endosomes and lysosomes and facilitate a Rab5–Rab7 hand-off (Fig. 6, inset) (26). GTP-bound Rab7 recruits the dynein/dynactin motor complex through the Rab interacting lysosomal protein (RILP) and in conjunction with Rabring7 promotes growth factor degradation (70, 71). Further work is, however, needed to determine precisely how the Rab7 CMT2B mutants may impact effector and regulatory protein interactions to fully understand the cause of disease pathology. The given examples underscore the importance of proper Rab regulation for maintaining normal cell physiology. Notably, changes in localization, increased or decreased activity, up- or downregulation can all contribute to disease. The exquisite tissue specificity of diseases that involve ubiquitously expressed proteins remains to be further explored, but suggests that targeted therapeutics could be a useful strategy.

## Endocytic Rab GTPases: therapeutic targets and precedents

Prospects for therapeutic intervention relevant to the described diseases have in many cases been reviewed separately. Here we focus primarily on interventions that target the Rab GTPases through modulation of membrane association, nucleotide binding or exchange, and inhibition of protein–protein interactions (Fig. 3) and include some limited description of interventions directed toward pathway interventions.

One strategy for inhibiting small GTPase function is to block membrane recruitment, through inhibition of protein prenylation (72). A limitation of this approach is the broad cellular importance of lipid modifications for the proper functioning of many GTPase families among other proteins and overlapping activity of prenylation inhibitors against farnesyl and geranylgeranyl transferases. Altering membrane recruitment of Rab GTPases has, however, shown particular selectivity and benefit in the area of bone metabolism. Examples here include NE10790 (a nitrogen-containing bisphosphonate) and zoledronic acid (73, 74). NE10790 was identified as a farnesyl diphosphate synthase inhibitor specific for osteoclasts and macrophages that blocked bone resorption and farnesylation of numerous small GTPases including Rab6 (73). The statin family of drugs that act by inhibiting the biosynthesis of isoprenoids has also been shown to have some efficacy against Rab GTPases (75). For example, mevastatin, which acts as non-specific reversible inhibitor of HMG-CoA reductase, blocked Rab5a targeting to transferrin receptor-positive early endosomes in HeLa cells (76). Statin use has, however, been linked to myopathy and rhabdomyolysis and thus, the quest for more specific prenylation inhibitors is actively moving forward (77). Even if not specific, the prenylation inhibitor results suggest that the strategy of delocalizing GTPases from membranes can be effective in some cases.

Another approach for modulating activation is through the inhibition of regulatory protein interactions. As proof-of-principle, the fungal metabolite brefeldin A has been successfully used as an inhibitor of ADP-ribosylation factor 1 (Arf1) GTPase (78, 79). Brefeldin A functions by targeting specific guanine GEFs, including Golgi complex-specific Brefeldin A resistance factor 1 (GBF1). A stable complex of Brefeldin A-GDP-Arf1-GEF on membranes prevents GDP/GTP exchange of Arf1 on membrane surfaces and disrupts Golgi morphology (80). Other Arf GTPase targeting molecules that may provide a platform for generating similar inhibitors for Rab GTPase GEFs are Exo2 and LG186 (81).

Among the Rho-family of GTPases, GEF inhibitors have been identified through screening and rational drug design, leading to a handful of Rho-family selective inhibitors (82). Thus far, there are no similar inhibitors of Rab GEFs. The growing list of known, specific Rab GEFs and GAPs together with growing structural information makes the targeting of Rab regulatory proteins through inhibitors or activators a tractable and worthy strategy. The therapeutic success and selectivity of competitive adenine nucleotide binding inhibitors for targeting tyrosine kinases and similar compounds for GTPases prompted our group to undertake a systematic screen for guanine nucleotide binding inhibitors. Using purified proteins immobilized on beads and assaying fluorescent GTP binding by flow cytometry we identified inhibitors selectively for Rho or Rab family GTPase members (83) (Agola et al., submitted). The versatility of flow cytometry measurements enabled the identification of a separate class of GTPase activators in the same screen (84). Activators might be useful for diseases where wild-type GTPase expression is reduced or there is altered activity, such as Niemann–Pick type C disease, Huntington's disease, Charcot-Marie-Tooth disease, and some cancers. Inhibitors of guanine nucleotide binding would be expected to block activation in cells, and with refinement to make them GTPase specific could offer unprecedented opportunities for modulating individual pathways in diseases where overexpression and hyperactivation of GTPases are a problem, e.g. cancers, neurodegenerative diseases, infectious diseases.

In some cases, targeting of associated signaling pathways rather than directly targeting the small GTPase may be beneficial. For example, valproic acid has been used to impact pathways linked to Rab5- and Rab7-regulated functions. Valproic acid is therapeutically used as a mood stabilizer for the treatment of bipolar disorder (85). Valproic acid is a histone deacetylase inhibitor that promotes neuronal differentiation and modulates signal-regulated kinase (ERK) and glycogen synthase kinase3*β* (GSK3*β*) pathways (86). In dorsal root ganglion neurons expressing a CMT2B-associated mutant Rab7, valproic acid was found to improve neurite formation via the c-Jun *N*-terminal kinase (87). Even though valproic acid does not target the GTPases directly, it is beneficial in CMT2B disease by altering gene expression and signaling.

Niemann–Pick type C disease is marked by the accumulation of unesterified cholesterol within the lysosomal storage organelles and inhibition of multiple Rab GTPases. Recently, 2-hydroxypropyl-*β*-cyclodextrin gained approval for treatment of Niemann–Pick type C disease (88). In human NPC1 and NPC2 mutant fibroblasts, treatment with 2-hydroxypropyl-*β*-cyclodextrin was reported to significantly reduce cholesterol levels in the storage organelles and relieve Rab GTPase inhibition (89). The systemic effects of cholesterol depletion are minimized during treatment of Niemann–Pick type C disease through cerebrospinal administration. On the contrary, protein aggregation underlying neurodegeneration is worsened by 2-hydroxypropyl-*β*-cyclodextrin derivatives, which would suggest caution in the use of *β*-cyclodextrin analogs to correct cholesterol homeostasis (90).

## Summary

The elucidation of the Rab GTPase circuitry that regulates endocytic membrane trafficking is proceeding at a rapid rate with a large literature that we have summarized in this review. Based on the highlighted studies, key themes emerge. Firstly, Rab GTPases function as scaffolds for the temporal and spatial regulation of cargo transport. Secondly, Rab GTPase functions are coordinately regulated through cascades that involve shared effectors and regulatory proteins and are responsive to cellular demand. Thirdly, Rab GTPase pathways interface hierarchically with other GTPases to integrate cell signaling and trafficking. Greater understanding of Rab GTPase functions has been gained through a combination of basic science and disease-based studies. Fourthly, dysregulated or mutant Rab GTPases and accessory proteins underlie many acquired and genetic human disease and is based on the many important functions Rab GTPases have in cellular and physiologic homeostasis. Foundational studies show that GTPases can be targeted by altering nucleotide binding or regulatory protein interactions with small molecules. Questions remain as to how tissue-specific pathologies arise from mutant GTPases that are ubiquitous and expressed in all cells. This may be due to redundant pathways or GTPase functions in some cell types, as well as tissue-specific effectors or regulators. As we move toward GTPase-targeted interventions and therapeutics, greater clarity in this arena will be essential. It will also be important to couple GTPase interventions with appropriate tissue-targeting strategies.
